# An Intuitive and Efficient Teleoperation Human–Robot Interface Based on a Wearable Myoelectric Armband

**DOI:** 10.3390/biomimetics10070464

**Published:** 2025-07-15

**Authors:** Long Wang, Zhangyi Chen, Songyuan Han, Yao Luo, Xiaoling Li, Yang Liu

**Affiliations:** 1 School of Mechanical Engineering, Xi’an Jiaotong University, Xi’an 710048, China; wangl521@stu.xjtu.edu.cn (L.W.); 3120101240@stu.xjtu.edu.cn (Z.C.); 3124301359@stu.xjtu.edu.cn (S.H.); luoyao45@stu.xjtu.edu.cn (Y.L.); 2 School of Art and Design, Xi’an University of Technology, Xi’an 710049, China; 105388@xaut.edu.cn

**Keywords:** wearable myoelectric armband, human–robot interaction, teleoperation, hybrid reference frame

## Abstract

Although artificial intelligence technologies have significantly enhanced autonomous robots’ capabilities in perception, decision-making, and planning, their autonomy may still fail when faced with complex, dynamic, or unpredictable environments. Therefore, it is critical to enable users to take over robot control in real-time and efficiently through teleoperation. The lightweight, wearable myoelectric armband, due to its portability and environmental robustness, provides a natural human–robot gesture interaction interface. However, current myoelectric teleoperation gesture control faces two major challenges: (1) poor intuitiveness due to visual-motor misalignment; and (2) low efficiency from discrete, single-degree-of-freedom control modes. To address these challenges, this study proposes an integrated myoelectric teleoperation interface. The interface integrates the following: (1) a novel hybrid reference frame aimed at effectively mitigating visual-motor misalignment; and (2) a finite state machine (FSM)-based control logic designed to enhance control efficiency and smoothness. Four experimental tasks were designed using different end-effectors (gripper/dexterous hand) and camera viewpoints (front/side view). Compared to benchmark methods, the proposed interface demonstrates significant advantages in task completion time, movement path efficiency, and subjective workload. This work demonstrates the potential of the proposed interface to significantly advance the practical application of wearable myoelectric sensors in human–robot interaction.

## 1. Introduction

The rapid development of artificial intelligence technologies has significantly enhanced the autonomous capabilities of robots in perception, decision-making, and planning [1], driving their widespread application in industries such as manufacturing [2,3], agriculture [4], healthcare [5,6], and household life [7]. However, even the most advanced autonomous control algorithms may still fail when confronted with complex and dynamic environments [8,9,10]. Therefore, the development of efficient and reliable human–robot interaction interfaces, which enable users to intervene in or fully take over robot control in real-time, has become crucial to ensuring the stable operation of autonomous systems [11]. Additionally, even when autonomous systems operate smoothly, providing direct control capabilities can enhance task flexibility, allowing users to seamlessly switch between fully autonomous, supervisory, or direct control modes, thereby improving the robot’s adaptability [12,13]. Therefore, designing a convenient and reliable human–robot interaction interface, especially one that enables non-expert users to achieve smooth on-demand control, is essential [14].

In recent years, significant advancements have been made in myoelectric gesture recognition technology, enabling accurate identification of a variety of gestures [15,16]. At the same time, with the rapid development of lightweight myoelectric armband devices (such as those from Meta [17], OYMotion [18], and FlectoThink [19]), myoelectric gesture interaction has attracted growing attention. Compared to visual recognition systems that are susceptible to environmental lighting and occlusion [20], as well as inertial capture systems that rely on continuous calibration and are cumbersome to wear [21], wearable lightweight myoelectric devices offer the following advantages: (1) lightweight and comfortable, worn like a smartwatch and available for use at any time; (2) strong environmental robustness, using muscle electrical signals for control, unaffected by external factors; (3) natural and simple operation, with predefined gestures enabling accurate control. Based on these advantages, myoelectric gesture interaction provides a natural and user-friendly robot control method, especially for non-expert users [22].

However, current research on myoelectric gesture recognition and interaction mainly focuses on improving the robustness of gesture recognition models to address the impact of electrode shift, limb movement, and other interferences on recognition accuracy [15,23,24]. There has been relatively little attention and in-depth exploration on how to intuitively and efficiently map recognized gestures to robot control commands, especially in scenarios that require teleoperation with visual feedback. Patricio et al. [25] utilized the Myo myoelectric armband to recognize arm movements for controlling the end-effector translation of the UR5 robot. Rotational control is achieved by selecting one of three predefined directions through recognized gestures, resulting in limited directional flexibility. Shin et al. [26] also employed the MYO armband to simultaneously control end-effector translation and rotation by recognizing arm movements. Control modes (translation and rotation) are switched through gestures. Kim et al. [27] utilized six gestures to control the rotation of the base, shoulder, and third joint of the wrist of the UR3 robot, respectively. Due to the control limitation of these three joints, the robot cannot fully maximize its capabilities. It is worth noting that these studies primarily focus on gesture recognition algorithms, using the recognized gestures to control robot movement in order to verify the feasibility of myoelectric gesture interaction, without delving into the intuitiveness and efficiency of the interaction. Additionally, these studies often assume that the user and the robot are in the same space and can directly observe the robot’s movements, without thoroughly analyzing the effects of visual feedback in teleoperation.

In practical application scenarios, robots are typically deployed in environments such as homes or factories, where users rely on the two-dimensional visual feedback provided by cameras to observe and control the robot [28]. When the user’s gesture intent is inconsistent in direction or meaning with the motion feedback of the robot displayed on the screen, a visual-motor misalignment issue arises, significantly reducing the intuitiveness of control [29]. The movement of the robot’s end-effector is usually based on a fixed base reference frame {B} mounted on the base, or a tool reference frame {T} that changes with the direction of the end-effector [30]. However, the motion observed by the user on the visual feedback screen is associated with the camera viewpoint, which differs from the motion direction in the {B} or {T} reference frames [31]. For example, in the {B} frame, the direction of robot translation is triggered by the same gesture changes, which changes with the camera viewpoint; in the {T} frame, the translation direction varies with the end-effector orientation. This inconsistency requires the user to perform mental rotation and cognitive adjustments, increasing the user’s operational burden and reducing efficiency [32,33,34]. Therefore, designing a control mapping mechanism that can alleviate or eliminate visual-motor misalignment is key to enhancing the intuitiveness of myoelectric gesture teleoperation. This challenge is well-documented in the broader teleoperation literature, and the use of control reference frames aligned with the robot’s base, tool, or the camera’s viewpoint is a recognized strategy for improving control intuitiveness [29,31,32]. However, the optimal mapping strategy for the unique constraints of myoelectric interfaces, which rely on a limited set of discrete gesture inputs, requires specific investigation.

In addition, the inefficiency of discrete control modes is another prominent issue. Currently, myoelectric gesture recognition often employs a “one gesture controls one degree of freedom” discrete control approach, where each gesture corresponds to the movement of the robot’s end-effector along a single axis [35,36]. However, for more complex movements (such as diagonal movement requiring the coordination of two degrees of freedom), the operator must frequently switch gestures to sequentially activate different degrees of freedom, which not only increases the robot’s movement path but also requires the operator to continuously maintain gestures to sustain the motion. This frequent switching in the single-degree-of-freedom control mode not only reduces efficiency but also leads to hand fatigue and a lack of smoothness. The myoelectric control applications mentioned earlier [25,26,27], while validating the feasibility of gesture recognition in driving robot motion, typically use basic discrete single-degree-of-freedom control and do not account for the inefficiencies introduced by this control mode.

To address the issues of poor intuitiveness caused by visual-motor misalignment and low efficiency resulting from discrete gesture control modes in myoelectric teleoperation, this study proposes a myoelectric teleoperation human–robot interaction interface based on a myoelectric armband. The interface integrates two core elements: (1) a hybrid reference frame that fully considers the camera viewpoint and end-effector characteristics to minimize visual-motor misalignment; (2) a control logic based on a finite state machine (FSM) to overcome the limitations of discrete gestures, enabling multi-degree-of-freedom coordination and speed variation, thereby improving overall operational efficiency and smoothness. Through multi-task experiments involving different end-effectors and camera viewpoints, the proposed integrated strategy has been validated to significantly improve the overall performance and user experience of myoelectric teleoperation.

## 2. Materials and Methods

### 2.1. Wearable Myoelectric Armband Interface

**Figure 1:**
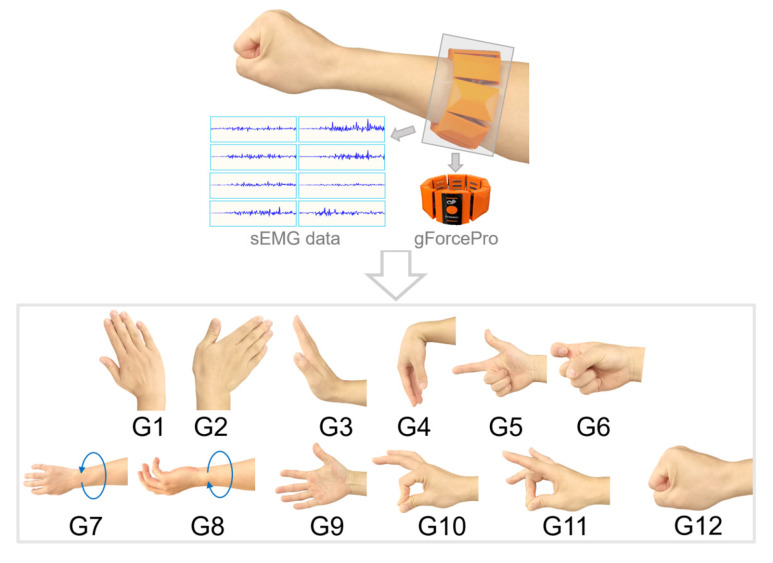
Wearable myoelectric armband gForcePro and the 12 recognized gestures.

This study employs a consumer-grade myoelectric armband sensor, the gForcePro, as the input interface for teleoperation. The device was used in its standard, off-the-shelf hardware configuration without any physical modifications to ensure replicability. As shown in the upper part of Figure 1, the armband is equipped with 8 channels of high-sensitivity myoelectric sensors with a sampling frequency of 200 Hz. Additionally, the armband integrates a button and a motor, supporting customizable button functions and motor vibration feedback. In previous work, we proposed a robust online myoelectric gesture recognition method based on this armband that integrates distribution shift detection and unsupervised domain adaptation [16]. This method is designed to automatically address various disturbances in myoelectric gesture interaction, eliminating the need for manual recalibration. Its reliability was validated in online experiments that simulated practical use, where combined interferences such as muscle fatigue and electrode shift were introduced. In these conditions, the model consistently maintained an average recognition accuracy of over 90% after a brief, automatic adaptation period. This high level of robustness ensures the reliability of gesture input for the teleoperation tasks described herein. Therefore, this study adopts the method and experimental process from [16] to train the gesture recognition model, identifying 12 gestures (G1–G12), as shown in the lower part of Figure 1. These gestures can be intuitively mapped to control commands to drive the robot to perform translational or rotational movements. For instance, gestures G1 and G2 trigger left–right translation, while G3 and G4 trigger up–down translation. This mapping fully leverages the characteristics of human muscle activity, making the operator’s control input more aligned with natural habits.

### 2.2. Hybrid Reference Frame Selection Based on Visual-Motor Misalignment Quantification

#### 2.2.1. Quantification of Visual-Motor Misalignment

**Figure 2:**
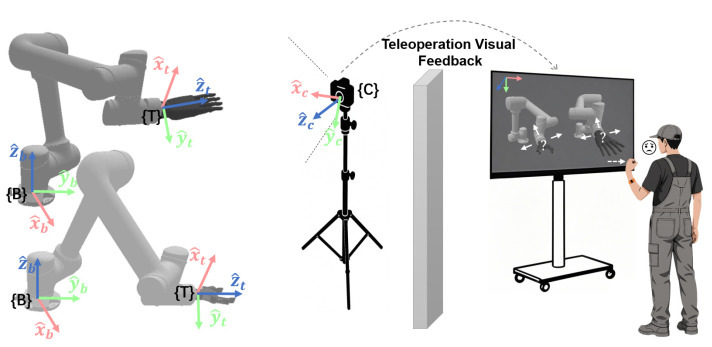
Diagram of visual-motor misalignment.

Visual-motor misalignment refers to the deviation between the expected motion, expressed by the operator’s gestures during teleoperation, and the actual motion observed on the screen. It is important to clarify that this concept addresses the directional consistency and intuitiveness of control, not the metrological positioning accuracy of the robotic system. As shown in Figure 2, if the control reference frame is not aligned with the screen view, it leads to visual-motor misalignment, where the motion direction shown on the screen deviates from the operator’s expectations. This inconsistency forces the operator to make additional cognitive adjustments, leading to frustration and increased cognitive load. Furthermore, this misalignment is further complicated by the differing motion characteristics of various end-effectors (e.g., dexterous hands or grippers). The misalignment between the expected motion and the actual motion is a key factor in determining the intuitiveness of human–robot interaction control [37]. To quantify this misalignment, this study introduces the misalignment metric M.

For translation, M is defined as the angle between the operator’s expected motion direction vector $v_{\exp}$ and the actual motion direction vector $v_{act}$, as observed on the screen.

(1) $$M={cos}^{-1}\left(\frac{v_{\exp}\cdot v_{act}}{\left\|v_{\exp}\right\|\left\|v_{act}\right\|}\right)$$

For rotation, M is defined as the angle between the operator’s expected rotation axis $a_{\exp}$ and the actual rotation axis $a_{\mathrm{act}}$, as observed on the screen.

(2) $$M={cos}^{-1}\left(\frac{a_{\exp}\cdot a_{act}}{\left\|a_{\exp}\right\|\left\|a_{act}\right\|}\right)$$

A smaller value of M indicates smaller misalignment, suggesting more intuitive control. Notably, this analysis focuses on the direction of motion, independent of speed.

#### 2.2.2. Analysis of Control Reference Frame Selection

This study uses the visual-motor misalignment metric M to evaluate the intuitiveness of three control reference frames (as shown in Figure 2) in both translation and rotation: the base reference frame {B} (fixed to the robot’s base), the tool reference frame {T} (which changes with the end-effector’s orientation), and the camera reference frame {C} (corresponding to the screen’s view). To calculate M, the operator’s expected motion must first be clearly defined. For translational motion in a typical vision-centric teleoperation scenario, the most intuitive expectation is movement along the screen’s perspective (i.e., the {C} frame). However, for rotational motion, the intuitive frame of reference is context-dependent: the user’s expectation may be anchored to the screen’s perspective (e.g., rotating an object in the view) or to the end-effector itself (e.g., a proprioceptive twisting action).

(1) Selection of Translational Reference Frame
  Taking gesture G2 as an example, suppose the intended motion produced by this gesture is the end-effector motion horizontally to the right on the screen, i.e., $v_{\exp}={\left[1,0,0\right]}^{T}$ (expressed in the {C} frame, representing the motion direction the operator expects to observe on the screen). The analysis of the three reference frames is as follows:
  (a) Base Reference Frame {B}
  Gesture G2 triggers the end-effector to translate along the $x_{b}$-axis of {B}, with the direction vector ${\left[1,\;0,\;0\right]}^{T}$ in the {B} frame. The actual motion direction feedback on the screen is $v_{\mathrm{act}}=R_{B}^{C}\cdot {\left[1,0,0\right]}^{T}$. $R_{B}^{C}$ represents the rotation matrix from {B} to {C} (obtained through hand–eye calibration). According to Equation (1), the misalignment metric is $M={cos}^{-1}\left({\left(R_{B}^{C}\right)}_{11}\right)$. Therefore, when the $x_{c}$-axis is aligned with the $x_{b}$-axis, M=0°. However, if the camera view deviates, for example, by rotating around the $y_{b}$-axis by an angle ($R_{B}^{C}=R_{y}\left(\theta \right)$), then ${\left(R_{B}^{C}\right)}_{11}=cos\theta$, i.e., $M=\left|\theta \right|>0{}^{\circ}$. This indicates that when {B} is chosen as the control reference frame, visual misalignment of translational motion will occur unless {C} is perfectly aligned with {B}.
  (b) Tool Reference Frame {T}
  Gesture G2 triggers the end-effector to translate along the $x_{t}$-axis of {T}, with the direction vector ${\left[1,\;0,\;0\right]}^{T}$ in the {T} frame. The actual motion direction is $v_{\mathrm{act}}=R_{B}^{C}\cdot$($R_{T}^{B}\cdot {\left[1,\;0,\;0\right]}^{T}$). $R_{T}^{B}$ is the robot pose matrix (obtained through the robot’s forward kinematics). The misalignment metric is $M={cos}^{-1}{\left(R_{B}^{C}R_{T}^{B}\right)}_{11}$. Since the orientation of {T} dynamically changes with the end-effector while the orientation of {C} is fixed, visual misalignment (i.e., M>0°) will occur unless the $x_{t}$-axis is aligned with the $x_{c}$-axis.
  (c) Camera Reference Frame {C}
  Gesture G2 triggers the end-effector to translate along the $x_{c}$-axis of {C}, with the direction vector ${\left[1,\;0,\;0\right]}^{T}$ in the {C} frame. In this case, $v_{\mathrm{act}}$ and $v_{\exp}$ coincide, i.e., M=0°. The axis of {C} is directly aligned with the screen’s directions (horizontal, vertical, depth), and no matter how the camera view changes, visual misalignment of translational motion will never occur.

**Figure 3:**
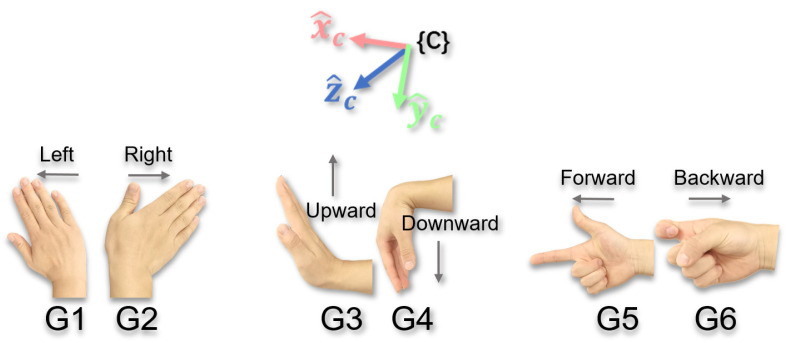
Schematic diagram of translation control gesture commands and control reference frame.

In summary, the camera reference frame {C} maintains M=0° for translation control under any viewpoint, with its visual consistency derived from direct alignment with the screen coordinate system. This makes it significantly more intuitive than {B} and {T} (which typically have M>0°). Therefore, binding translation commands to {C} is the ideal choice for translation tasks. Specifically, this includes the following: left–right translation (G1/G2, along the positive/negative $x_{c}$-direction), up–down translation (G3/G4, along the positive/negative $y_{c}$-direction), forward–backward translation (G5/G6, along the positive/negative $z_{c}$-direction), as shown in Figure 3.

(2) Selection of Rotational Reference Frame
  Taking gestures G7/G8 (triggering roll) as an example, the user’s expectations may be based on the screen perspective (e.g., around the $z_{c}$-axis of {C}) or the end-effector itself (e.g., around the $z_{t}$-axis of {T}), depending on the task requirements and the type of end-effector. The analysis of the three reference frames is as follows:
  (a) Base Reference Frame {B}
  Gesture G7 or G8 triggers the end-effector to rotate around the $z_{b}$-axis of {B}. The rotation axis in {B} is ${\left[0,0,1\right]}^{T}$. The actual rotation axis feedback on the screen is $a_{\mathrm{act}}=R_{B}^{C}\cdot {\left[0,\;0,\;1\right]}^{T}$. If the expected rotation is around the screen’s depth direction, then $a_{\exp}={\left[0,\;0,\;1\right]}^{T}$. According to Equation (2), the misalignment metric is $M={cos}^{-1}{\left(R_{B}^{C}\right)}_{33}$. Unless the $z_{b}$-axis is aligned with the $z_{c}$-axis, M>0°. If the expected rotation is around the end-effector’s $z_{t}$-axis, $a_{\exp}={R_{B}^{C}\cdot R_{T}^{B}\cdot \left[0,\;0,\;1\right]}^{T}$, and obviously M>0°, unless the $z_{t}$-axis is aligned with the $z_{b}$-axis. Therefore, the fixed nature of {B} makes it difficult to adapt to changes in viewpoint or end-effector orientation, and the rotation feedback often deviates from the expected direction.
  (b) Tool Reference Frame {T}
  Gesture G7 or G8 triggers the end-effector to rotate around the $z_{t}$-axis of {T}. The rotation axis in {T} is ${\left[0,0,1\right]}^{T}$. The actual rotation axis is $a_{act}={R_{B}^{C}\cdot R_{T}^{B}\cdot \left[0,\;0,\;1\right]}^{T}$. If the expected rotation is around the screen depth direction, $a_{\exp}={\left[0,\;0,\;1\right]}^{T}$, then $M={cos}^{-1}\left({\left(R_{B}^{C}R_{T}^{B}\right)}_{33}\right)$. Typically, M>0°, unless the $z_{t}$-axis is aligned with the $z_{c}$-axis. If the expected rotation is around the end-effector’s $z_{t}$-axis, then ${a_{\exp}=a}_{\mathrm{act}}$, so M=0°. Therefore, {T} is intuitive for local rotations of the end-effector, but may lead to misalignment in tasks based on the screen perspective.
  (c) Camera Reference Frame {C}
  Gesture G7 or G8 triggers the end-effector to rotate around the $z_{c}$-axis of {C}, i.e., $a_{\mathrm{act}}={\left[0,\;0,\;1\right]}^{T}$. If the expected rotation is around the screen depth direction, ${a_{\exp}=a}_{\mathrm{act}}$, then M=0°. If the expected rotation is around the end-effector’s $z_{t}$-axis, $a_{\exp}={R_{B}^{C}\cdot R_{T}^{B}\cdot \left[0,0,1\right]}^{T}$, then $M={cos}^{-1}\left({\left(R_{B}^{C}R_{T}^{B}\right)}_{33}\right)$. Unless the $z_{t}$-axis is aligned with the $z_{c}$-axis, M>0°. Therefore, {C} is intuitive in tasks dominated by the screen perspective, but may not be suitable for local rotations of the end-effector.

**Figure 4:**
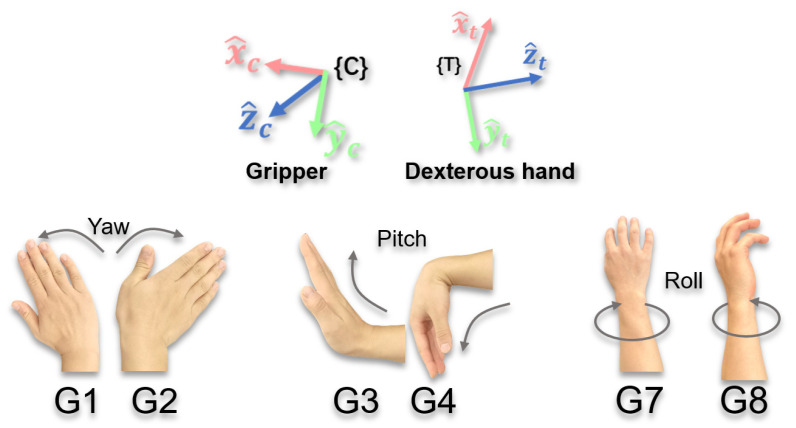
Schematic diagram of rotational control gesture commands and control reference frames.

In summary, the choice of rotation reference frame depends on the type of end-effector and the task requirements. When the end-effector is a gripper, due to its simple structure and lack of orientation, it is primarily used for grasping and releasing tasks, where its rotational requirements focus on overall posture adjustment, and consistency with the screen perspective is more important. Hence, binding to {C} (with M=0°) aligns better with the visual-first principle. However, for a dexterous hand, its motion patterns resemble those of the human hand. For example, wrist flexion and extension movements (G3 and G4) naturally map to pitch motion about the $y_{t}$-axis, while forearm rotation movements (G7 and G8) naturally map to roll around the $z_{t}$-axis. Hence, binding to {T} (with local M=0°) is more intuitive due to semantic alignment. In summary, a hybrid strategy is used for rotation tasks: bind grippers to {C} and dexterous hands to {T}. Consequently, rotational commands are defined as G1/G2 for yaw, G3/G4 for pitch, and G7/G8 for roll, as shown in Figure 4.

Furthermore, it is worth noting that to achieve comprehensive control over all six degrees of freedom (6-DOF) of the end-effector using a limited and easy-to-memorize set of gestures, this study employs a function overloading strategy. As shown in Figure 3 and Figure 4, the functions of several core gestures (i.e., G1–G4) are mode-dependent. The operator can switch between translational and rotational motion modes using a dedicated auxiliary gesture, with the mechanics detailed in Section 2.3.

#### 2.2.3. Hybrid Reference Frame Design

Based on the previous analysis, this study proposes a hybrid reference frame to optimize the intuitiveness of myoelectric interaction. For translational motion, bind to the camera reference frame {C}. For rotational motion, dynamically select based on the type of end-effector: if the end-effector is a gripper, bind to {C} and adjust according to the screen angle; if the end-effector is a dexterous hand, bind to {T} to align with the local semantics of the end-effector.

Given the initial end-effector pose is

(3) $$T_{T}^{B}=\left[\begin{array}{cc} R_{T}^{B} & p^{B}\\ 0 & 1 \end{array}\right]$$

The following is the calculation process for the updated robot pose ${T^{\prime }}_{T}^{B}$ after applying the gesture commands.

**Figure 5:**
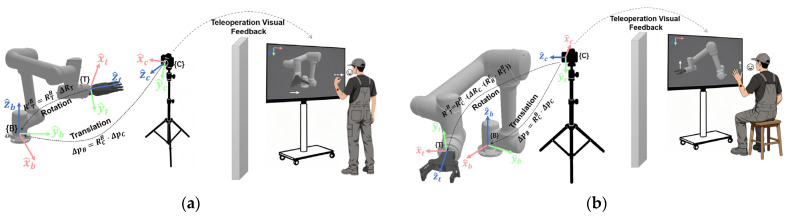
The human–robot interaction diagram of the proposed hybrid reference frame: (**a**) the end-effector is a dexterous hand, with translation bound to the {C} frame and rotation bound to the {T} frame; (**b**) the end-effector is a gripper, with both translation and rotation bound to the {C} frame.

For translation motion control, as shown in Figure 5, the displacement increment $\Delta p_{C}={\left[\Delta x_{C},\Delta y_{C},\Delta z_{C}\right]}^{T}$ generated in the {C} frame is transformed into the {B} frame as follows $\Delta p_{B}=R_{C}^{B}\cdot \Delta p_{C}$. The updated position is $q_{B}^{\prime }=q_{B}+\Delta p_{B}$.

For rotational motion control, the reference frame is selected based on the end-effector type, and the end-effector pose is updated using the incremental rotation matrix. Specifically, for a dexterous hand (bind to {T}), as shown in Figure 5a, the orientation is updated as ${R^{\prime }}_{T}^{B}=R_{T}^{B}\cdot \Delta R_{T}$, where $\Delta R_{T}=R_{x_{T}}\left(\theta_{x}\right)R_{y_{T}}\left(\theta_{y}\right)R_{z_{T}}\left(\theta_{z}\right)$; for a gripper (bind to {C}), as shown in Figure 5b, the orientation is updated as ${R^{\prime }}_{T}^{B}$ = $R_{C}^{B}\cdot (\Delta R_{C}\cdot \left(R_{B}^{C}\cdot R_{T}^{B}\right))$, where $\Delta R_{C}=R_{x_{C}}\left(\theta_{x}\right)R_{y_{C}}\left(\theta_{y}\right)R_{z_{C}}\left(\theta_{z}\right)$.

The translation velocity (i.e., $\Delta x_{C},\Delta y_{C},\Delta z_{C}$) and rotation velocity (i.e., $\theta_{x},\theta_{y}\theta_{z}$) are adjusted according to the finite state machine (see Section 2.3.2). In summary, through the proposed hybrid reference frame, the end-effector pose is updated as

(4) $${T^{\prime }}_{T}^{B}=\left[\begin{array}{cc} {R^{\prime }}_{T}^{B} & q^{\prime B}\\ 0 & 1 \end{array}\right]$$

### 2.3. Finite State Machine-Based Motion Control Logic

Although the proposed hybrid reference frame enhances the intuitiveness of human–robot interaction, the discrete nature of myoelectric gestures leads to inefficient interaction. Common control modes typically map each gesture to a single degree of freedom, requiring the operator to continuously maintain the gesture. This results in frequent gesture switching when performing multi-degree-of-freedom tasks, which not only reduces efficiency but also leads to rapid hand fatigue. Furthermore, the fixed motion speed further limits the system’s adaptability. To fully exploit the potential of myoelectric control, this study proposes a motion control logic based on a finite state machine (FSM), aiming to achieve the following functionalities:

(1) Multi-mode switching: Provide multiple operation modes (e.g., translation/rotation, coarse/fine adjustments) to suit different task scenarios and reduce the physical workload on the operator.
(2) Multi-degree-of-freedom cooperative control: Support multi-degree-of-freedom motion of the end-effector through gesture combinations, enhancing operational coherence and efficiency.
(3) Dynamic speed adjustment: Allow operators to adjust motion speed in real-time according to task requirements, increasing control flexibility and adaptability.

The proposed FSM implements modular control through four distinct states—Idle, Translate (Progressive Coarse Translation), Rotate (Progressive Coarse Rotation), and FineTune (Continuous Fine Adjustment for Translation/Rotation).

#### 2.3.1. Gesture Function Classification and Motion Mode Definition

Myoelectric gestures G1–G11 are classified into three categories based on their control objectives:

Translation Gestures (G1–G6): Control the translation of the end-effector along the axes of the selected reference frame, as shown in Figure 3.

Rotation Gestures (G1–G4, G7, and G8): Control the rotation of the end-effector around the axes of the selected reference frame, as shown in Figure 4.

**Figure 6:**
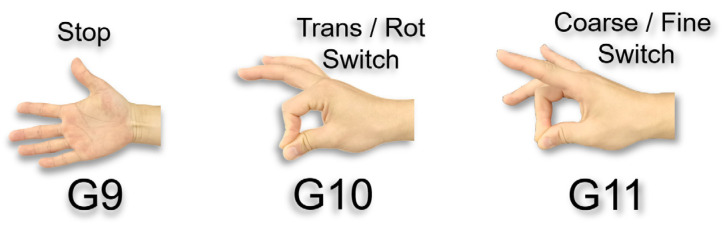
Schematic diagram of auxiliary gestures.

Auxiliary Gestures: G9 (Pause motion), G10 (Switch motion mode: Translation/Rotation), G11 (Switch control mode: Coarse/Fine adjustment), as shown in Figure 6.

The system defines two motion modes:

(1) Translational Motion Mode: Adjusts the end-effector’s position using gestures G1–G6.
(2) Rotational Motion Mode: Adjusts the end-effector’s orientation using gestures G1–G4 and G7–G8.

Each motion mode supports two control modes:

(1) Progressive Coarse Adjustment: After executing a single gesture command, the robot sustains motion after a single gesture, freeing the operator from continuously holding the pose. During this phase, multi-degree-of-freedom synthesis and dynamic speed adjustment are supported (repeated gestures accelerate the movement, while reverse gestures decelerate it). This mode is suitable for controlling the end-effector’s rapid, large-range movements.
(2) Continuous Fine Adjustment: The gesture continuously drives the end-effector along a single degree of freedom, with the movement stopping when the gesture is released. The speed remains fixed, making it suitable for small-range, high-precision adjustments.

#### 2.3.2. Finite State Machine Design

**Figure 7:**
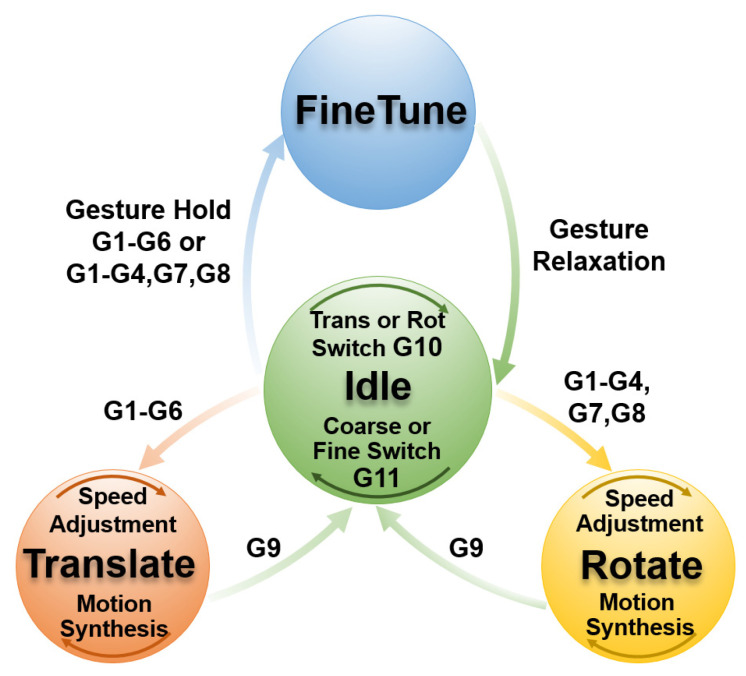
The transition rules and operational mechanisms for each state of the proposed finite state machine.

The FSM starts in the Idle state and implements flexible state transitions and motion control based on gesture input and the current mode (Motion Mode: Translation/Rotation; Control Mode: Coarse Adjustment/Fine Adjustment). As shown in Figure 7, the transition rules and operational mechanisms for each state are as follows:

(1) Idle (Initial State)
  Description:
  The system is in its initial or paused state, and the end-effector remains stationary.
  Parameters:
  Default motion mode: Translational Mode
  Default control mode: Fine Adjustment Mode
  Operational Mechanism:
  (a) The end-effector remains stationary and serves as a hub for mode switching and state transitions.
  (b) G10 and G11 adjust the current mode to prepare for subsequent state transitions
  Transition Rules:
  G9: Remains in the Idle state.
  G10: Switches the motion mode (Translation/Rotation).
  G11: Switches the control mode (Coarse Adjustment/Fine Adjustment).
  (a) If the current mode is Coarse Translation: G1–G6 transitions to the Translate state.
  (b) If the current mode is Coarse Rotation: G1–G4, G7–G8 transitions to the Rotate state.
  (c) If the current mode is Fine Translation: G1–G6 transitions to the FineTune state.
  (d) If the current mode is Fine Rotation: G1–G4, G7–G8 transitions to the FineTune state.
(2) Translate (Progressive Coarse Translation State)
  Description:
  The end-effector translates along the axes of the camera reference frame {C}, supporting multi-degree-of-freedom synthesis and speed adjustments.
  Parameters:
  Translation increment: $\Delta p_{C}=(\Delta x_{C}$, $\Delta y_{C}$, $\Delta z_{C})$.
  Base step size: $\Delta p_{0}$.
  Operational Mechanism:
  (a) Multi-degree-of-freedom synthesis: Different gestures overlay to generate composite motion. For example, G1 followed by G3 results in $\;\Delta p_{C}$ = ($\Delta p_{0}$,$\;\Delta p_{0}$, 0).
  (b) Speed adjustment: Repeated gestures accelerate the movement (e.g., G1 followed by G1 results in $\Delta p_{C}$ = ($2\Delta p_{0}$, 0, 0)), while reverse gestures decelerate it (e.g., G1 followed by G1 then G2 results in $\Delta p_{C}$ = ($\Delta p_{0}$, 0, 0)).
  (c) Motion update: $q_{B}^{\prime }=q_{B}+R_{C}^{B}\cdot \Delta p_{C}$.
    Transition Rules:
    G1–G6: Self-loop, adjust translation speed, and motion composition.
    G9: Return to the Idle state and maintain the translational coarse adjustment mode.
(3) Rotate (Progressive Coarse Rotation State)
  Description:
  The end-effector rotates along the axes of the selected reference frame ({C} or {T}), supporting multi-degree-of-freedom motion synthesis and speed adjustments.
  Parameters:
  Rotation increment: $\theta_{x}$, $\theta_{y}$, $\theta_{z}$.
  Base step size: $\theta_{0}$.
  Operational Mechanism:
  (a) Multi-degree-of-freedom synthesis and speed adjustment logic is similar to that of Translate.
  (b) Motion update: Gripper (using {C} frame) is ${R^{\prime }}_{T}^{B}$=$R_{C}^{B}\cdot (\Delta R_{C}\cdot \left(R_{B}^{C}\cdot R_{T}^{B}\right))$. Dexterous Hand (using {T} frame) is ${R^{\prime }}_{T}^{B}=R_{T}^{B}\cdot \Delta R_{T}$.
  Transition Rules:
  G1–G4, G7–G8: Self-loop, adjust rotation speed, and motion composition.
  G9: Return to the Idle state and maintain the rotation coarse adjustment mode.
(4) FineTune (Continuous Fine Adjustment State)
  Description:
  The gesture continuously drives the end-effector’s movement along a single degree of freedom, and motion ceases upon gesture release.
  Parameters:
  Motion speed: Fixed small step size ($\Delta p_{0}$ and $\theta_{0}$).
  Operational Mechanism:
  Movement continues as long as the gesture is held, and stops once the gesture is released.
  Transition Rules:
  Based on the motion mode from the Idle state (Fine Translation or Fine Rotation), apply G1–G6 or G1–G4, G7–G8 gestures.

It is important to note that direct transitions between the Translate, Rotate, and FineTune states are not allowed. The system must first return to the Idle state, where transitions between states can then occur. This design ensures a hierarchical distinction between coarse and fine adjustments, enhancing the clarity of the control logic.

#### 2.3.3. Feedback Mechanism

The feedback mechanism is crucial for enhancing user interaction experience and system robustness. Particularly considering that this study employs a gesture function overloading strategy (e.g., G1–G4 can be used for both translation and rotation), clear state feedback is key to avoiding “mode errors,” where the user might confuse the current control mode. Therefore, this study employs both vibration and visual feedback to provide the operator with real-time FSM state information, ensuring accuracy and safety during operation.

Vibration feedback: When the system switches motion modes (e.g., triggered by gesture G10), the vibration motor in the myoelectric armband emits a single vibration lasting 0.5 s to notify the operator that the mode has been successfully changed.

Visual feedback: A simple interface has been developed to display the camera feedback and the current state of the FSM, including Fine Translation, Fine Rotation, Coarse Translation, and Coarse Rotation, ensuring that the operator can accurately monitor the system status.

## 3. Experiments

### 3.1. Experimental Setup

The experiment utilizes the gForcePro myoelectric armband from Oymotion (Shanghai, China) as the gesture input device. The robot employs a six-degree-of-freedom collaborative robotic arm, RM65, from Realman Robotics (Beijing, China). The end-effectors include a gripper, EG2-4B, and a dexterous hand, RH56BFX, both from Inspire Robots (Beijing, China). The camera used is from Hikvision (Hangzhou, China).

**Figure 8:**
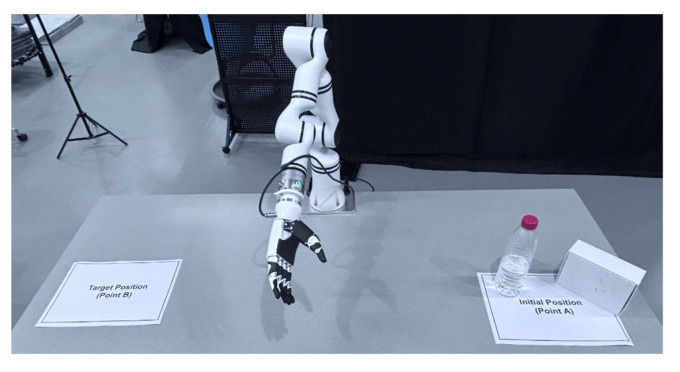
Schematic diagram of experimental setup.

The scene layout is shown in Figure 8. Before each task, the robot is set to a standardized initial pose. The spatial relationship between the robot’s coordinate frames and the camera was determined as follows: the robot’s end-effector pose (tool frame {T} relative to base frame {B}) is continuously updated via its forward kinematics, while the fixed transformation between the camera frame {C} and the robot base frame {B} was determined using a standard hand–eye calibration procedure prior to the experiments. This calibration allows for the accurate transformation of control commands between reference frames. The operator is required to control the robot to reach the initial position (Point A), grasp the target object (such as a box or bottle), and then place the object at the target position (Point B). The distance between Point A and Point B is sufficiently large so that the robot cannot reach it solely through translation and must perform rotational motion, ensuring that the operator performs a combination of translational and rotational control. The base translational speed of the robot in this experiment is set to 0.02 m/s, and the rotational speed is set to 0.08 rad/s. Since the control loop frequency of the RM65 robot is 50 Hz, the base step size is calculated as follows: $\Delta p_{0}=0.02\;m/s\times 0.02\;s=0.0004\;m$ and $\theta_{0}=0.08\;rad/s\times 0.02\;s=0.0016\;rad$. It is important to note that this study focuses on the intuitiveness and efficiency of the control interface, rather than challenges related to long-distance teleoperation. Therefore, all teleoperation experiments were conducted within a single local area network, where communication latencies are minimal. Although inherent delays are introduced by processes such as myoelectric signal acquisition, wireless transmission, and gesture recognition computation, experimental results demonstrate that they were consistently low and did not perceptibly affect control fluency.

### 3.2. Experimental Tasks

**Figure 9:**
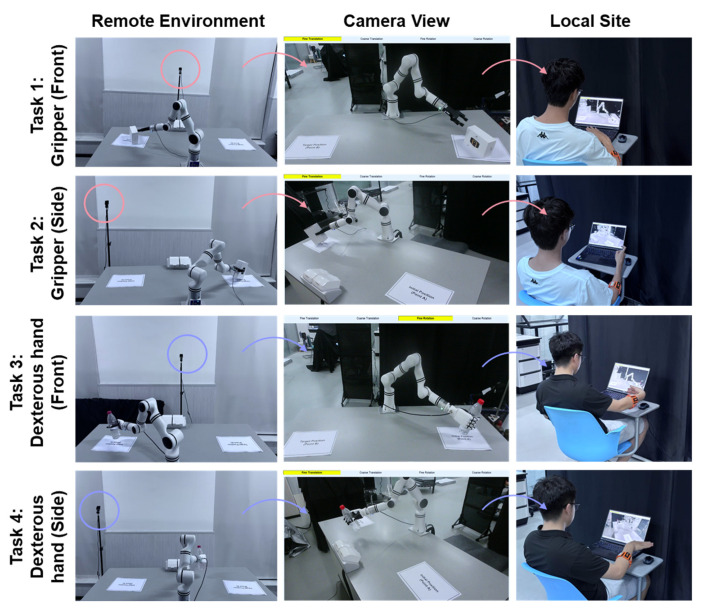
Schematic diagram of the four tasks.

To evaluate the performance of different control strategies under various conditions (including different end-effectors and camera perspectives), four pick-and-place tasks were designed, as shown in Figure 9:

Task 1 (Gripper, Front View): Using a gripper, pick up a box from point A and place it in the point B area, with the camera perspective in front of the robot.

Task 2 (Gripper, Side View): Similar to Task 1, but with the camera perspective at the robot’s left front side.

Task 3 (Dexterous Hand, Front View): Using a dexterous hand, pick up a bottle from point A and place it in the point B area, with the camera perspective in front of the robot.

Task 4 (Dexterous Hand, Side View): Similar to Task 3, but with the camera perspective at the robot’s left front side.

### 3.3. Comparison Methods

Hybrid_All: The proposed myoelectric teleoperation interface, which includes the hybrid reference frame (translation bound to the camera reference frame {C}, rotation bound to {C} or {T} depending on the end-effector type) and FSM control logic.

Hybrid_Discrete: The same hybrid reference frame as Hybrid_All, but without the FSM control logic. This method is used to isolate and evaluate the contribution of the FSM control logic. Specifically, gestures G1-G6 continuously control the translation directions of the end-effector, gestures G1-G4 and G7-G8 continuously control rotation, and gesture G10 is used to switch between translation and rotation motion modes.

Camera: Both translation and rotation controls are relative to the camera reference frame {C}, with the gesture control logic being the same as that of Hybrid_Discrete.

Base: Both translation and rotation controls are relative to the robot base reference frame {B}, with the gesture control logic being the same as that of Hybrid_Discrete.

Tool: Both translation and rotation controls are relative to the tool reference frame {T}, with the gesture control logic being the same as that of Hybrid_Discrete.

### 3.4. Participants

Fifteen healthy participants (9 males, 6 females, aged 25.3 ± 3.1 years) were recruited to participate in the experiments for Tasks 1 to 4. All participants were right-handed with normal or corrected-to-normal vision. Among them, 10 participants reported having some experience with robotic operation or experience with complex video games, while the remaining 5 participants were novices without such experience. Before the experiment, all participants were thoroughly informed about the purpose, procedure, potential risks, and signed an informed consent form. The experimental protocol was approved by the Ethics Committee of Xi’an Jiaotong University, China.

### 3.5. Evaluation Metrics

#### 3.5.1. Objective Metrics

(1) Task Completion Time (s): The total time from when the operator issues the first valid control command to when the item is successfully placed in the target area. A shorter time indicates higher operational efficiency.
(2) Translational Path (m): The cumulative translation distance traveled by the end-effector in three-dimensional space during the task. A shorter path generally indicates more precise control, fewer redundant movements, and higher intuitiveness.
(3) Rotational Path (rad): The cumulative rotation angle of the end-effector’s orientation during the task. A smaller value indicates more efficient and direct posture adjustments.

#### 3.5.2. Subjective Metrics

NASA-TLX Score (TLX Overall, 0–100): After completing each task, participants were required to fill out the NASA-TLX questionnaire. The questionnaire included six dimensions: Mental Demand (MD), Physical Demand (PD), Temporal Demand (TD), Performance (P), Effort (E), and Frustration Level (F). The scores of the six TLX subscales were averaged with equal weighting to calculate the overall TLX score. A lower score indicated a lower perceived task load, suggesting that the system was easier to use and the user experience was better.

### 3.6. Experimental Procedure

#### 3.6.1. Preparation Phase

After each participant wore the myoelectric armband, the gesture recognition model was trained following the experimental procedure described in the literature [16]. This model was capable of recognizing 12 gestures, with G1-G11 used for FSM and G12 used for controlling the opening and closing of the end-effector.

#### 3.6.2. Training Phase

(1) Experiment Introduction: The purpose, procedure, and evaluation metrics of the experiment were thoroughly explained to the participants.
(2) Gesture Practice: Participants were guided to practice the execution of gestures for at least 5 min, enabling them to perform the 12 gestures proficiently.
(3) Control Method Practice: Participants were instructed to practice robot motion control for at least 10 min using the methods described in Section 3.3, familiarizing themselves with the operational process of each method.

#### 3.6.3. Execution Phase

Each participant was required to complete Tasks 1–4 using all five control methods. To eliminate potential learning effects and sequence bias on the experimental results, the presentation order of the five control methods was balanced among participants using a Latin Square Design. For each control method, each task was repeated 3 times. The system automatically recorded the objective metric data for each trial. After completing all four tasks (3 repetitions × 4 tasks = 12 trials) under a given control method, participants took a short break and immediately filled out a NASA-TLX questionnaire for that control method. One-way analysis of variance (ANOVA) was used to compare the performance differences in the different control methods across each evaluation metric.

## 4. Results

**Figure 10:**
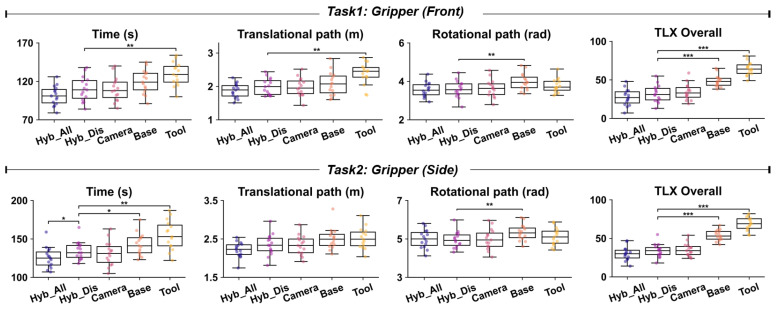

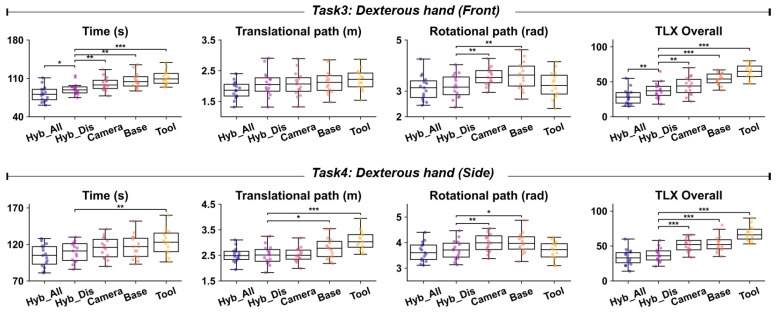
The objective and subjective performance of each method in Tasks 1–4 (* *p* < 0.1, ** *p* < 0.05, *** *p* < 0.01).

The experimental results are shown in Table 1 and Figure 10. Overall, the Hybrid_All method consistently outperforms Hybrid_Discrete, Camera, Base, and Tool across all evaluation metrics for Tasks 1–4. The differences between the method groups are highly statistically significant across various evaluation metrics (with most metrics showing large F-values, small *p*-values, and large effect sizes η^2^), indicating that these performance differences have substantial practical significance. A detailed analysis of the experimental results is presented as follows.

### 4.1. Evaluation of the Intuitiveness Advantage of the Hybrid Reference Frame (Hybrid_Discrete vs. Camera, Base, Tool)

To verify the contribution of the hybrid reference frame in enhancing operational intuitiveness, Hybrid_Discrete (which includes only the hybrid reference frame, without FSM) was compared with the discrete control methods based on three fixed reference frames: Camera, Base, and Tool.

Comparison with Camera: In the gripper tasks (Tasks 1 and 2), the performance (time, path) of Hybrid_Discrete and Camera was similar, and the post hoc test confirmed no significant difference between them (Tukey’s HSD, *p* > 0.05 for both metrics), which was expected. In this configuration, both methods bind translation and rotation to the camera reference frame {C}, providing an interactive experience that is highly consistent with visual feedback. This suggests that for simple end-effectors like the gripper, the camera reference frame {C} already provides an intuitive control experience. However, in the more complex dexterous hand tasks (Tasks 3 and 4), significant performance differences were observed. Although the translational paths remained similar (as translation is still bound to {C}), the Camera method performed significantly worse than Hybrid_Discrete in terms of rotational path and task completion time. This finding directly validates our analysis in Section 2.2.2, which predicts that binding dexterous hand rotation to {C} introduces significant visual-motor misalignment (M>0°) because the on-screen rotation does not match the hand’s natural, posture-relative motion. For example, in Task 3, Camera took 100.1 s with 3.57 rad of rotation, while Hybrid_Discrete took 90.9 s with 3.2 rad of rotation. These differences were marginally significant (Tukey’s HSD, *p* < 0.1 for both metrics), suggesting a trend towards the superiority of the hybrid reference frame when binding the tool reference frame {T} for dexterous hand rotations. Relying solely on {C} for fine posture adjustments with the dexterous hand is not intuitive, leading the operator to spend more time adjusting the posture. This is supported by the subjective cognitive load data (NASA-TLX). In the dexterous hand tasks, the Camera method yielded higher TLX scores than Hybrid_Discrete (e.g., in Task 3, Camera: 44.3, Hybrid_Discrete: 37.6), a difference that also approached statistical significance (Tukey’s HSD, *p* < 0.1) and pointed to higher mental demand and frustration.

Comparison with Base: Compared to Hybrid_Discrete, the Base method showed a clear pattern of poorer performance across all tasks. While not every metric was statistically significant in every task, post hoc tests revealed a consistent trend where key indicators like task completion time and path length were frequently and significantly worse. This outcome is a direct empirical validation of the high visual-motor misalignment (M>0°) predicted for the {B} frame in our theoretical analysis (Section 2.2.2). Since {B} is fixed, when the camera perspective or end-effector orientation changes, there is a high likelihood of visual-motor misalignment between the operator’s gesture intention and the screen feedback. This forces the operator to frequently perform mental rotations and directional corrections, which reduces efficiency and increases cognitive load. The higher NASA-TLX scores for the Base method (e.g., in Task 4, Base scored 53.8, while Hybrid_Discrete scored 37.3, a highly significant difference with Tukey’s HSD, *p* < 0.01) also confirm its higher operational load.

Comparison with Tool: The Tool method primarily performed poorly in translation control, resulting in longer translational distances and task completion times compared to Hybrid_Discrete. For instance, in the comparison of translational path for Tasks 1 and 4, the Tool method was significantly worse (Tukey’s HSD, *p* < 0.05). This finding aligns perfectly with the theoretical prediction from Section 2.2.2, where translation in the {T} frame was shown to produce significant visual-motor misalignment (M>0°) unless the tool’s axes happen to align with the camera’s axes. Because the translation direction in {T} changes with the end-effector’s orientation, the resulting motion observed by the user is often unpredictable and counter-intuitive from the screen’s perspective. However, in terms of rotation control, the performance of Tool was similar to Hybrid_Discrete (especially in dexterous hand tasks), indicating that when the rotation semantics are strongly correlated with the end-effector’s own posture (e.g., the self-spin of the dexterous hand), {T} provides intuitive control (corresponding to M=0°). Nevertheless, the non-intuitive nature of translation in {T} significantly affected the overall performance and user experience, with NASA-TLX scores being generally and significantly higher (Tukey’s HSD, *p* < 0.05).

The above comparisons demonstrate the intuitiveness of the hybrid reference frame: by consistently binding translation to the camera reference frame {C} and dynamically selecting {C} or {T} for rotation based on the end-effector’s characteristics, Hybrid_Discrete significantly alleviates visual-motor misalignment, enhances operational intuitiveness, and reduces the user’s subjective cognitive load.

### 4.2. Evaluation of the Efficiency of FSM Control Logic (Hybrid_All vs. Hybrid_Discrete)

The contribution of the FSM control logic in enhancing operational efficiency and smoothness was evaluated by comparing Hybrid_All with Hybrid_Discrete.

**Figure 11:**
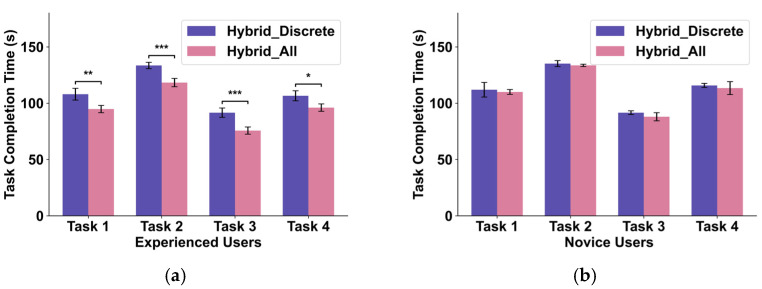
Comparison of task completion time based on user experience level, (**a**) experienced group, (**b**) novice group (* *p* < 0.1, ** *p* < 0.05, *** *p* < 0.01).

Objective Metric Analysis: Although the differences in translational and rotational paths between Hybrid_All and Hybrid_Discrete were generally not statistically significant, a consistent trend was observed where Hybrid_All reduced task completion time. However, this trend did not achieve strong statistical significance across all tasks, such as Task 1 and Task 4, which did not show significant differences. We hypothesized that this was due to varying levels of user experience. To investigate this, we performed a more granular analysis by segmenting the participants into an “experienced group” (n = 10) and a “novice group” (n = 5). The results, presented in Figure 11, reveal a clear distinction: for experienced users (Figure 11a), the efficiency benefit of the FSM was significant. This group was able to leverage the advanced features of the FSM to achieve a statistically significant reduction in task completion time across all four tasks. For novice users (Figure 11b), there was no significant difference in completion time between the two methods. Observation of their behavior revealed that novices tended to use the Hybrid_All method more cautiously, often defaulting to simpler, single-degree-of-freedom commands, which made their behavior functionally similar to using the Hybrid_Discrete mode. This breakdown strongly supports our hypothesis: the FSM provides a measurable efficiency gain for users who can quickly overcome a small learning curve, and the initial, weaker overall result was a direct consequence of averaging these two distinct user groups. Beyond just measuring if users were faster, we analyzed how they used the different control modes offered by the FSM to validate its design. We analyzed the system logs to see which control mode—“Progressive Coarse Adjustment” or “Continuous Fine Adjustment”—users preferred during different phases of the task. The analysis revealed a clear and rational pattern of strategic mode switching: for the initial phase of each trial, where the robot had to be moved from its starting position toward the target, users overwhelmingly chose the “Progressive Coarse Adjustment” mode. This allowed them to cover large distances rapidly and with minimal physical effort (i.e., without having to constantly hold a gesture). As the robot’s end-effector entered the immediate vicinity of the target, a distinct shift in user strategy was observed. Here, users switched to and predominantly used the “Continuous Fine Adjustment” mode for the delicate final alignment and grasping maneuvers. These behaviors provide evidence that the dual-mode (Coarse/Fine) control structure of the FSM is an intuitive tool that operators naturally and strategically employ to meet the demands of the task.

Subjective Metric Analysis: Although the objective performance gains were experience-dependent, Hybrid_All consistently achieved lower average NASA-TLX scores across all tasks and for both user groups compared to Hybrid_Discrete. This suggests that even when novice users did not fully leverage the FSM for speed, the availability of features like “Progressive Coarse Adjustment” (reducing the need to continuously hold a gesture) and clear mode switching significantly lowered their perceived physical demand, effort, and frustration, thereby improving the overall user experience.

### 4.3. Analysis of the Impact of Camera Perspective and End-Effector Type

The experimental results reveal the significant impact of camera viewpoint and end-effector type on performance, validating the advantages of our hybrid framework design, as demonstrated in Video S1: Hybrid_All Demonstration.

First, the consistent performance degradation in side-view tasks (Tasks 2 and 4) emphasizes the intrinsic cognitive load of non-frontal perspectives, which introduce visual distortion and ambiguous depth perception. The camera-centric translation of the proposed hybrid framework proves essential in mitigating this issue. By ensuring that user gestures in the semantic direction always correspond to the same movement on-screen, the framework provides a stable and predictable anchor. While this does not eliminate the inherent difficulty of a side view, it significantly reduces control errors and user frustration compared to the severely misaligned Base or Tool frames.

Second, the choice of end-effector is also a key factor determining the efficiency of myoelectric gesture-based teleoperation. For the dexterous hand, binding its rotation to the tool frame {T} creates a powerful proprioceptive and semantic mapping. Operators control the hand as if it were an extension of their own body, leveraging natural wrist flexion/extension (pitch) and forearm rotation (roll) gestures. The hand’s anthropomorphic form provides immediate, unambiguous visual feedback about its own state (e.g., palm orientation), making it inherently intuitive for the operator to know which gesture to apply next. Conversely, for the structurally simple gripper, which lacks such strong anthropomorphic cues, a user’s rotational intent is more abstract and aligns more naturally with the 2D plane of the screen. By binding its rotation to the camera frame {C}, our method directly maps gestures to on-screen rotations (e.g., a “twist” gesture causes the “gripper to rotate clockwise along the screen’s depth direction”), providing a more direct and predictable interaction method for such tools.

## 5. Discussion

### 5.1. Advantages of the Proposed Method

The portability and environmental robustness of lightweight myoelectric sensors theoretically enable natural and intuitive teleoperation. This study addresses the key barriers to its practical deployment—namely, the inherent visual-motor misalignment in teleoperation and the inefficiency of discrete gesture control. While our approach is built upon the well-established principle of coordinate transformation between reference frames, its primary contribution lies in the systematic analysis, application, and integration of these principles into a cohesive interface specifically tailored for myoelectric control. Experimental results demonstrate that the proposed interface significantly outperforms benchmark methods in both objective performance metrics (task completion time and movement path efficiency) and subjective workload assessments (NASA-TLX), primarily due to the combined contribution of its two core components.

First, the design of the hybrid reference frame based on the quantification of visual-motor misalignment enhances the intuitiveness of operation. By binding translation control to the camera reference frame {C}, which most closely aligns with the user’s visual perception, the cognitive load induced by visual-motor misalignment is effectively alleviated. The dynamic adaptation of the hybrid reference frame for rotational control (gripper bound to {C}, dexterous hand bound to {T}) has also been proven effective, accommodating the characteristics of different end-effectors and task requirements. Additionally, this study also reveals the challenges posed by the camera viewpoint, with the side view significantly increasing operational difficulty and user workload compared to the front view. This highlights the critical role of a robust and intuitive control reference framework in maintaining the usability and user comfort of myoelectric gesture-based teleoperation under non-ideal visual conditions.

Second, the introduction of FSM control logic significantly enhances operational efficiency and smoothness. These efficiency gains are most significant for experienced users, who quickly learn to leverage multi-degree-of-freedom coordination and progressive speed adjustments. This is further supported by the analysis of control mode usage, which revealed a clear strategic shift from ‘Progressive Coarse Adjustment’ for rapid, large-scale movements to ‘Continuous Fine Adjustment’ for precise terminal positioning, validating the FSM design philosophy. Importantly, even for novices, the FSM demonstrably reduces subjective workload (NASA-TLX scores). This is likely due to features like progressive motion (eliminating the need to constantly hold a gesture), which reduce physical fatigue and frustration, enhancing the overall subjective experience even if objective task time is not immediately improved.

### 5.2. Limitations and Future Work

Although the proposed myoelectric teleoperation interface demonstrates significant advantages, there are still some limitations that need to be addressed in future work.

First, regarding the choice of the hybrid reference frame, this study still relies on the researcher’s manually specified expected movements and does not fully capture the user’s true intentions. Future research could explore reference frame selection mechanisms that are more aligned with user intentions. For example, an interactive user selection mechanism could be introduced, allowing users to directly specify their movement expectations (e.g., “move right along the screen” or “rotate around its own axis”) through a simple interface. Subsequently, the system will automatically select the optimal reference frame based on the reference frame selection process proposed in this study, which is guided by visual-motor misalignment indicators.

Second, the efficiency advantages of FSM are somewhat dependent on the user’s proficiency, and there is a certain learning curve for novices. To make the benefits of the FSM more broadly applicable, future work should focus on developing methods to shorten this learning phase. For instance, future studies could explore interactive, FSM-guided tutorials that provide real-time feedback and progressively introduce advanced features like multi-degree-of-freedom coordination. Another promising direction is to create personalized control modes that adapt to a user’s demonstrated skill level, perhaps starting with a simplified ‘novice mode’ and unlocking more complex functionalities as proficiency is demonstrated, thereby helping novices quickly gain confidence and master the system.

Third, while function overloading of gestures (G1–G4) enhances control compactness by reducing the total number of gestures a user must learn, it introduces a potential risk of mode errors. This occurs when the operator loses track of the current active mode (e.g., Translation or Rotation) and inadvertently executes an incorrect command. Our interface mitigates this risk through clear vibrotactile and visual feedback upon every mode switch, ensuring the operator maintains situational awareness. Despite these mitigation measures, the need for the user to explicitly track and switch modes still imposes a cognitive burden, highlighting an inherent limitation of the design. Exploring alternative mode-switching mechanisms, or even context-aware automatic mode switching for high-stress or safety-critical scenarios, could be a valuable direction for future work.

Fourth, while the experimental tasks effectively demonstrated our method’s advantages, they were conducted in a relatively simple environment. Real-world applications often involve more dynamic and cluttered spaces, where the operator’s cognitive load would increase and challenges like collision avoidance would become prominent. A key direction for future work is to integrate semi-autonomous features, such as sensor-based collision avoidance, with our current framework. This would allow the operator to leverage our intuitive interface for high-level directional control while the system autonomously manages low-level safety, enhancing the system’s robustness and practical utility for complex, real-world tasks.

## 6. Conclusions

This study proposes an integrated myoelectric teleoperation interface aimed at addressing the issues of low intuitiveness and inefficiency caused by visual-motor misalignment and discrete gesture control in myoelectric teleoperation. The interface combines an intuitive hybrid reference frame with an efficient FSM-based control logic. Experimental results demonstrate that, compared to benchmark methods, the proposed interface significantly enhances operational intuitiveness and efficiency, effectively reducing users’ cognitive and physical workload. This work provides support for developing more natural and efficient myoelectric teleoperation human–robot interaction interfaces, contributing to enhancing the potential of wearable myoelectric sensors in the field of teleoperation robot control.

## Figures and Tables

**Table 1:** Statistical results of the evaluation metrics for each method in Tasks 1–4.

Measures	Hybrid_All Mean (SD)	Hybrid _Dis Mean (SD)	Camera Mean (SD)	Base Mean (SD)	Tool Mean (SD)	*F*(4, 70)	*p*	*η* ^2^
Task 1: Gripper (Front)								
Time (s)	101.4 (12.6)	110.1 (16.1)	110.0 (15.8)	119.5 (15.5)	129.7 (15.2)	7.67	<0.001	0.31
Translational path (m)	1.87 (0.21)	1.99 (0.25)	1.98 (0.27)	2.08 (0.35)	2.37 (0.33)	6.58	<0.001	0.27
Rotational path (rad)	3.57 (0.38)	3.60 (0.43)	3.62 (0.46)	3.98 (0.43)	3.78 (0.36)	2.51	0.051	0.13
TLX Overall	27.4 (10.9)	32.6 (11.2)	34.4 (10.6)	48.7 (7.7)	64.5 (8.5)	35.22	<0.001	0.67
Task 2: Gripper (Side)								
Time (s)	125.6 (13.6)	134.7 (11.9)	131.1 (15.9)	142.9 (14.3)	154.9 (19.1)	8.51	<0.001	0.33
Translational path (m)	2.22 (0.20)	2.35 (0.28)	2.33 (0.26)	2.51 (0.28)	2.52 (0.27)	3.55	0.011	0.17
Rotational path (rad)	5.00 (0.48)	4.96 (0.43)	4.99 (0.54)	5.33 (0.42)	5.12 (0.46)	1.64	0.173	0.12
TLX Overall	30.2 (8.9)	34.0 (8.8)	35.3 (8.4)	53.9 (6.9)	68.7 (8.7)	57.38	<0.001	0.77
Task 3: Dexterous hand (Front)								
Time (s)	81.8 (14.1)	90.9 (10.9)	100.1 (12.6)	106.1 (13.8)	110.9 (13.2)	12.31	<0.001	0.41
Translational path (m)	1.87 (0.29)	2.08 (0.42)	2.07 (0.41)	2.14 (0.38)	2.20 (0.34)	1.71	0.157	0.11
Rotational path (rad)	3.13 (0.49)	3.20 (0.46)	3.57 (0.35)	3.62 (0.55)	3.25 (0.51)	3.24	0.017	0.16
TLX Overall	28.8 (11.2)	37.6 (11.6)	44.3 (13.3)	54.0 (8.3)	64.7 (10.8)	23.58	<0.001	0.57
Task 4: Dexterous hand (Side)								
Time (s)	104.7 (15.2)	109.5 (13.4)	115.6 (14.8)	116.5 (16.4)	123.3 (17.6)	3.13	0.021	0.15
Translational path (m)	2.52 (0.29)	2.51 (0.37)	2.53 (0.29)	2.79 (0.43)	3.09 (0.38)	7.6	<0.001	0.31
Rotational path (rad)	3.64 (0.38)	3.72 (0.38)	4.00 (0.37)	3.99 (0.40)	3.68 (0.35)	3.34	0.015	0.16
TLX Overall	33.8 (11.5)	37.3 (10.1)	51.6 (9.6)	53.8 (12.2)	67.9 (10.6)	24.15	<0.001	0.58

## Data Availability

The datasets related to this study are available from the corresponding author upon reasonable request.

## Supplementary Materials

The following supporting information can be downloaded at: https://www.mdpi.com/article/10.3390/biomimetics10070464/s1, Video S1: Hybrid_All Demonstration.
